# Use of Cabozantinib in a Patient With *EGFR*-Mutated Non–Small-Cell Lung Cancer Harboring Acquired *CCDC6-RET* Fusion

**DOI:** 10.1200/PO.18.00295

**Published:** 2019-05-03

**Authors:** Young Kwang Chae, Pedro Viveiros, Caio T. Heleno, Haris Bilal, Bhoomika A. Sukhadia, Michael S. Oh, Muhamad Mubbashir Sheikh, Wade T. Iams, Lee Chun Park

**Affiliations:** ^1^ Northwestern University, Chicago, IL; ^2^ Vanderbilt University, Nashville, TN; ^3^ Kosin University, Pusan, South Korea

## INTRODUCTION

Genomic evaluation has allowed for the detection of targetable mutations in tumor samples and can now be applied to cell-free circulating tumor DNA (ctDNA).^1^ Development of targeted therapies has transformed the landscape of lung cancer treatment. Actionable mutations in non–small-cell lung cancer (NSCLC) include activating mutations in epidermal growth factor receptor (*EGFR*), which denotes a subset of patients with lung cancer who are profoundly sensitive to tyrosine kinase inhibitors (TKIs). However, *EGFR*-mutated NSCLC often develops acquired resistance to TKIs.^2-4^ Recently, a clinical trial demonstrated an increase in progression-free survival and overall survival when comparing osimertinib with first-generation TKIs.^5^ Because it has become a first-line treatment option for *EGFR*-mutated NSCLC, resistance to osimertinib is a major concern that may become more prevalent in the following years.^6^

NSCLC often develops resistance to treatment via mechanisms that are poorly understood.^6^ Here, we report an acquired *CCDC6-RET* rearrangement in a patient with *EGFR*-mutated (T790M) NSCLC whose disease progressed during osimertinib treatment. This is a rare case of an acquired *CCDC6-RET* rearrangement associated with resistance to osimertinib. The identification of this rearrangement guided the decision to use cabozantinib, a RET inhibitor approved for medullary thyroid carcinoma and renal cell carcinoma.^7,8^

## CASE REPORT

A 46-year-old woman, who was a never-smoker, initially presented with cough and hoarseness for 2 months. Computed tomography (CT) showed a right lower lobe mass with involvement of hilar, mediastinal, paratracheal, and bilateral supraclavicular lymph nodes. Positron emission tomography–CT identified the corresponding hypermetabolic lesions. Subcarinal lymph node biopsy confirmed the diagnosis of adenocarcinoma with *EGFR* exon 19 deletion. The patient was started on afatinib and tolerated it well.

**FIG 1.**
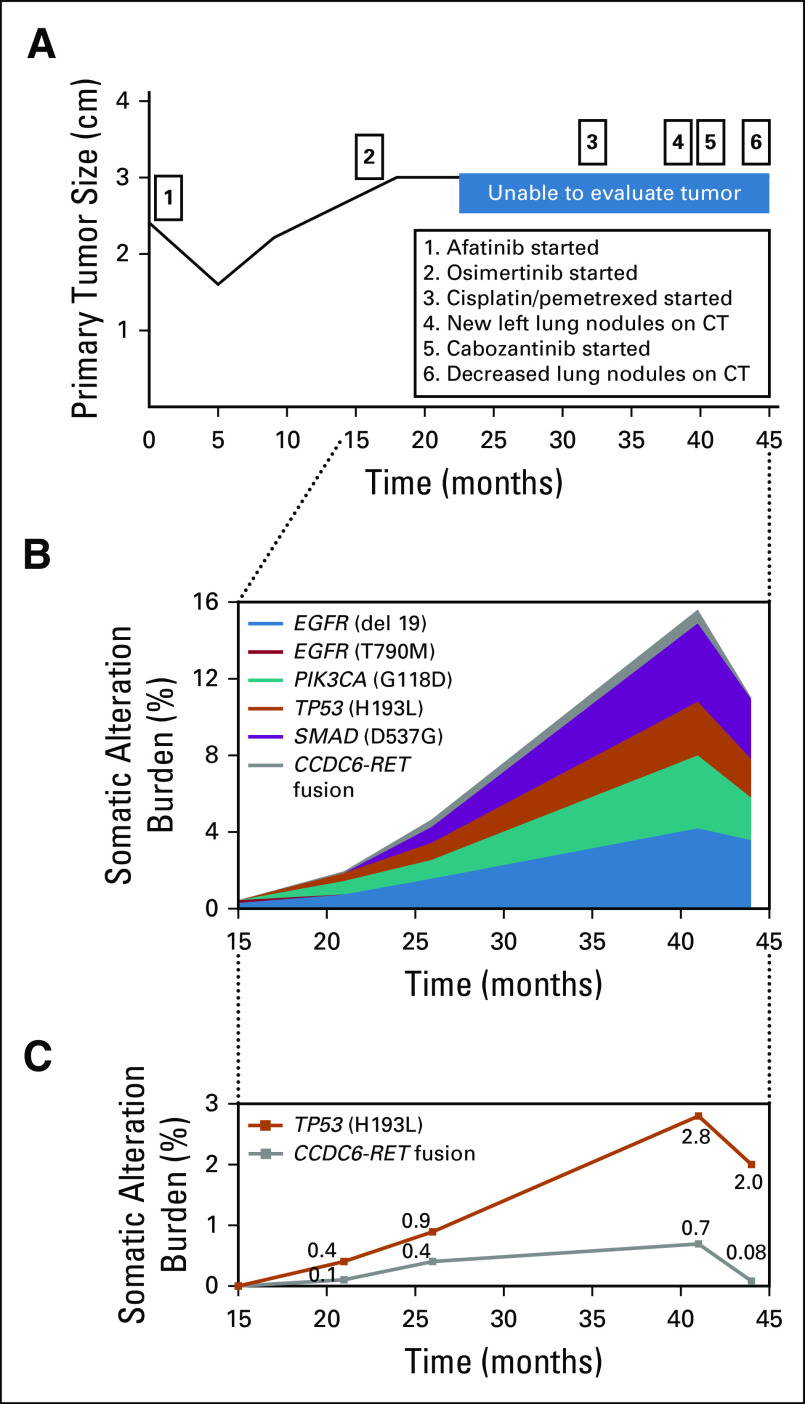
(A) Timeline depicting changes in tumoral size, treatment lines, and progression of disease events. (B) Next-generation sequencing (NGS) of circulating tumor DNA depicting somatic mutation burden of primary and acquired mutations. The tumor response mapping illustrates the mutant allele percentage (percent cell-free DNA [cfDNA]) of observed somatic variants at each sample submission time point. The somatic alteration burden value refers to the maximum percentage cfDNA detected at each time point and has been used to illustrate how tumor progeny is responding to the selective pressure of treatment. Note that this graph depicts each variant allele’s frequency complementarily, meaning that the percentage shown corresponds to the sum of all variant alleles’ frequency at a given time point. (C) Illustration of NGS selectively highlighting molecular response of *CCDC6-RET* fusion and *TP53* to cabozantinib.

A CT scan 4 months later showed partial response (Fig 1A). Nine months after initiation of therapy, however, the patient displayed growth of the known right lung lesion as well as a new subcentimeter nodule in the right lower lobe. Genomic profiling with ctDNA next-generation sequencing (NGS; Guardant Health, Redwood City, CA) identified an *EGFR* T790M mutation with a variant allele frequency of 0.2% (Fig 1B)

The patient was then started on osimertinib. CT scans after 7 months showed a new right-sided pleural effusion, with an associated dense consolidation that was considered to represent either atelectasis or therapy-related pneumonitis. This consolidation obscured her primary tumor, which no longer could be followed radiographically. Cytology of fluid from thoracentesis was positive for malignancy. Repeat ctDNA NGS no longer detected the T790M mutation but presented new mutations in *PIK3CA* (G118D, 0.7%) and *TP53* (H193L, 0.4%) as well as a *CCDC6-RET* rearrangement with a frequency of 0.1% (Fig 1C). At that point, patient was offered platinum-based chemotherapy but refused it. Instead, she opted to continue osimertinib with the addition of bevacizumab.

Fourteen months from the start of osimertinib, the patient had recurrent malignant effusions with possible pleural-based metastases concerning for disease progression. A new evaluation of ctDNA NGS still did not detect *EGFR* T790M mutation but showed an increase in the frequency of *CCDC6-RET* rearrangement from 0.1% to 0.4% (Fig 1C). A new biopsy from pleural tissue detected no programmed death ligand 1 expression in immune cells and an expression as low as 1% in tumor cells. Therefore, the patient started receiving standard-of-care chemotherapy with cisplatin and pemetrexed. She completed six cycles of chemotherapy with partial response followed by seven cycles of pemetrexed maintenance. Approximately 10 months after the start of chemotherapy, imaging revealed new left-sided lung nodules alongside low-volume liver metastases consistent with progression of disease.

Evaluation of ctDNA NGS detected additional increase in the frequency of *CCDC6-RET* rearrangement to 0.7% (Fig 1C). The patient was thus started on cabozantinib 60 mg/d. She developed grade 1 palmar-plantar erythrodysesthesia as an adverse effect, but otherwise tolerated the treatment well. After 2 months of treatment, ctDNA NGS detected a lower level of *CCDC6-RET* rearrangement (0.08%; Fig 1C). Three months after cabozantinib initiation, CT imaging still could not visualize her primary tumor but demonstrated decreased burden of bilateral pulmonary nodules as well as a discrete increase in pre-existent liver metastases. The patient reported marked improvement of dyspnea and pain as well as increased activity tolerance.

## DISCUSSION

We report emergence of *CCDC6-RET* rearrangement in the setting of osimertinib use in a patient with *EGFR* T790M–mutated NSCLC. The development of third-generation EGFR TKIs has been a major breakthrough in the treatment of cancers with T790M mutation. However, tumor cells develop resistance to these newer EGFR inhibitors by mechanisms that are less clear. The C797S *EGFR* mutation is detected in ctDNA in nearly 40% of these patients and has been implicated in osimertinib resistance.^9-12^ Other less commonly reported mutations include *MET*, *ERBB2* (human epidermal growth factor receptor), *BRAF*, *EGFR* (L718Q), *KRAS*, and *G12S*.^6,13-16^ Many of these occurred with the loss of *EGFR* T790M mutation, suggesting that T790M-positive clones were suppressed, but T790M wild-type cells with other driver mutations were able to mediate resistance.^6^ Epithelial-mesenchymal transition changes and small-cell transformation have also been reported in some cases of resistance. Nonetheless, in approximately 15% to 20% of the cases, the mechanism of acquired resistance to EGFR TKIs is yet to be discovered.

In this patient, we identified an uncommon acquired *CCDC6-RET* rearrangement that is also found in approximately 1% to 2% of NSCLCs at primary diagnosis.^17^ This rearrangement raises interest as a result of its possible role in resistance, emerging under the selective pressure of treatment. Fusion of the RET tyrosine kinase domain with an upstream coiled-coil domain has been shown to promote self-dimerization, resulting in constitutive signaling via prosurvival and proliferative pathways.^18^

*CCDC6-RET* rearrangements accounted for 23% of genetic alterations in a recent international registry of *RET*-rearranged lung cancers.^19^ At the initial diagnosis, de novo *RET* rearrangements have been considered to be mutually exclusive with other common driver mutations, such as those in *EGFR*, *BRAF*, *KRAS*, and *ALK*.^17^ Trials assessing clinical and pathologic characteristics of these de novo *RET* rearrangement suggest increased frequency in nonsmokers and patients with poorly differentiated adenocarcinomas, solid subtype, younger age, Asian origin, or small tumors (3 cm or smaller) with N2 disease.^17,20,21^ Germline gain-of-function mutations in *RET* predispose carriers to multiple endocrine neoplasia type 2, whereas somatic gain-of-function *RET* mutations have been commonly reported in sporadic medullary thyroid cancer and papillary thyroid cancer.^22,23^

Several commercially available multikinase inhibitors, such as vandetanib, cabozantinib, sorafenib, sunitinib, lenvatinib, and ponatinib, have shown activity against RET kinase (Table 1). Cabozantinib inhibits a broad range of tyrosine kinases, including RET, VEGFR2, mesenchymal-epithelial transition, AXL receptor tyrosine kinase, and *KIT* proto-oncogene receptor tyrosine kinase (c-KIT).^24-26^ Furthermore, several selective RET inhibitors are currently under clinical investigation. One example is LOXO-292, which had a good toxicity profile and showed activity against *RET*-mutated or translocated tumors, including those with previous resistance to multikinase inhibitors (Table 2).

**TABLE 1.**
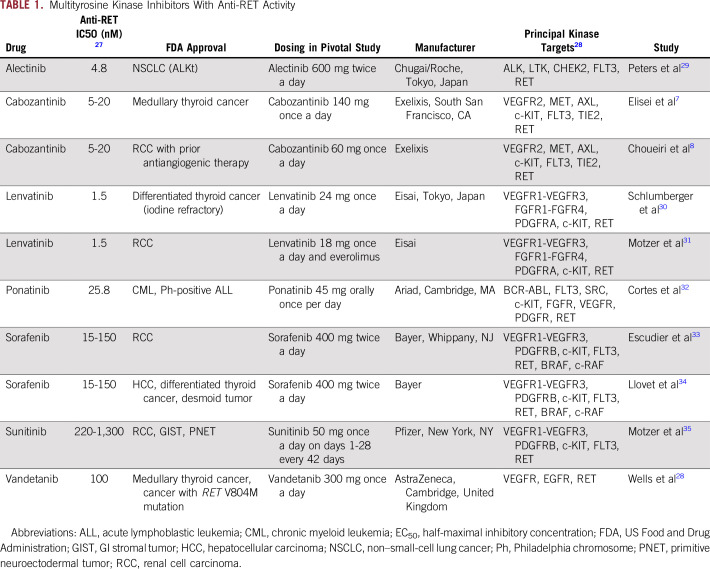
Multityrosine Kinase Inhibitors With Anti-RET Activity

**TABLE 2.**
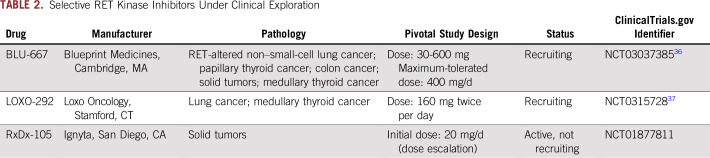
Selective RET Kinase Inhibitors Under Clinical Exploration

Cabozantinib is recommended by the National Comprehensive Cancer Network for treatment of medullary thyroid and clear cell renal cancers. Its use in *RET*-mutated tumors is approved by the US Food and Drug Administration and has been reported in a phase II clinical trial of patients with NSCLC.^24^ In this trial, an overall response rate of 28% was reported with a favorable safety profile. In our patient, cabozantinib was administered at a starting dose of 60 mg orally once per day, as recommended in the National Comprehensive Cancer Network guidelines. This dose has comparable plasma exposure (area under the plasma concentration-time curve) as the US Food and Drug Administration–approved dose of 140 mg per day used for the treatment of patients with metastatic medullary thyroid carcinoma.^19,24,38^ After 3 months of cabozantinib use in our patient, there was a reduction in ctDNA level of *CCDC6-RET* rearrangement and significant improvement in reported symptoms. ctDNA has been useful in demonstrating molecular response without the need for tissue biopsies and is a convenient way to assess for driver mutations and molecular response.^39^ Absolute levels of ctDNA have also been significantly correlated with tumor volume measured by CT and positron emission tomography–CT imaging.^40,41^ However, it has so far been used as an exploratory measure of response. Clinical and imaging data remain sovereign.

It is still unclear whether the presence of a rare yet targetable mutation represents a putative resistance mechanism. For this patient, the *RET* rearrangement was possibly not the only mechanism of acquired resistance. Loss of T790M coincided with the emergence of several mutations in oncogenic drivers, although none have been proven to be associated with osimertinib resistance. To date, we do not have approval on drugs that act on the other potential targets such as *PIK3CA*. Hence, a basket trial with enrollment across multiple centers may be needed to accrue the sufficient number of patients to generate robust data. Incorporating adaptive trial designs can also allow newly identified driver mutations to be added as the search for actionable targets continues. Furthermore, retrospective data sets can complement clinical trial results, as exemplified in a recently published global registry of RET-directed treatment outcomes.^19^

In conclusion, we report a case of acquired *CCDC6-RET* rearrangement in a patient with *EGFR-*mutated NSCLC. Treatment with the RET inhibitor cabozantinib led to significant clinical improvement and associated reduction in levels of *CCDC6-RET* detected from ctDNA. This suggests cabozantinib could be explored as a potential treatment strategy in NSCLC with *CCDC6-RET* rearrangement.
